# Association of prognostic nutritional index with risk of contrast induced nephropathy: A meta-analysis

**DOI:** 10.3389/fnut.2023.1154409

**Published:** 2023-03-23

**Authors:** Wei-Ting Chang, Cheuk-Kwan Sun, Jheng-Yan Wu, Po-Yu Huang, Ting-Hui Liu, Ying-Jen Chang, Yao-Tsung Lin, Fu-Chi Kang, Kuo-Chuan Hung

**Affiliations:** ^1^Division of Cardiology, Department of Internal Medicine, Chi-Mei Medical Center, Tainan City, Taiwan; ^2^Department of Biotechnology, Southern Taiwan University of Science and Technology, Tainan City, Taiwan; ^3^College of Medicine, Institute of Clinical Medicine, National Cheng Kung University, Tainan City, Taiwan; ^4^Department of Emergency Medicine, E-Da Hospital, I-Shou University, Kaohsiung City, Taiwan; ^5^School of Medicine for International Students, College of Medicine, I-Shou University, Kaohsiung City, Taiwan; ^6^Department of Nutrition, Chi Mei Medical Center, Tainan City, Taiwan; ^7^Department of Internal Medicine, Chi Mei Medical Center, Tainan City, Taiwan; ^8^Department of General Internal Medicine, Chi Mei Medical Center, Tainan City, Taiwan; ^9^Department of Anesthesiology, Chi Mei Medical Center, Tainan City, Taiwan; ^10^Department of Anesthesiology, Chi Mei Medical Center, Chiali, Tainan City, Taiwan; ^11^School of Medicine, College of Medicine, National Sun Yat-sen University, Kaohsiung City, Taiwan

**Keywords:** prognostic nutritional index, contrast-induced nephropathy, percutaneous coronary intervention, myocardial infarction, coronary angiography

## Abstract

**Background:**

Although prognostic nutritional index (PNI) has been frequently applied in patients with malignancy or those during postoperative recovery, whether it is also an optimal indicator of the risk of contrast-induced nephropathy (CIN) in patients receiving coronary angiography remains uncertain. This meta-analysis aimed at investigating the clinical association of PNI with the risk of CIN in patients receiving coronary angiography or percutaneous coronary intervention.

**Methods:**

Embase, Medline, Cochrane Library, and Google scholar were searched for studies until January 2023. The relationship between CIN risk and PNI (i.e., low vs. high) (primary outcome) as well as other variables (secondary outcomes) were analyzed using a random-effects model.

**Results:**

Overall, 10 observational studies with 17,590 patients (pooled incidence of CIN: 18%) were eligible for analysis. There was a higher risk of CIN in patients with a low PNI compared to those with a high PNI [odd ratio (OR) = 3.362, 95% confidence interval (CI): 2.054 to 5.505, *p* < 0.0001, *I*^2^ = 89.6%, seven studies, 12,972 patients, certainty of evidence: very low]. Consistently, a lower PNI was noted in patients with CIN compared to those without (Mean difference = −5.1, 95% CI: −6.87 to −3.33, *p* < 0.00001, *I*^2^ = 96%, eight studies, 15,516 patients, certainty of evidence: very low). Other risks of CIN included diabetes and hypertension, while male gender and the use of statins were associated with a lower risk of CIN. Patients with CIN were older, had a higher creatinine level, and received a higher contrast volume compared to those without. On the other hand, pre-procedural albumin, estimated glomerular filtration rate, ejection fraction, hemoglobin, lymphocyte ratio were found to be lower in patients with CIN than in those without.

**Conclusion:**

This meta-analysis highlighted an inverse association of PNI with the risk of CIN, which required further studies for verification.

**Systematic review registration:**

[https://www.crd.york.ac.uk/prospero/], identifier [CRD42023389185].

## 1. Introduction

The advancements of coronary interventions have significantly improved the survival of patients with coronary arterial disease (CAD), especially those with acute coronary syndrome (ACS) (1, 2). However, the exposure of contrast media during coronary intervention may cause kidney injury and contrast-induced nephropathy (CIN), thereby increasing the risks of hospitalization and even mortality (3–5). Previous studies indicated an incidence of CIN around 9% and that of renal failure requiring hemodialysis up to 0.5% (6). Several risk factors including older age, hemodynamic instability, pre-existing renal failure, diabetes mellitus (DM), heart failure, and the volume of contrast media have been reported to be linked to the development of CIN (3). On the other hand, malnutrition, which is commonly observed in critically ill patients and known to tightly correlate with acute kidney injury, has not been well studied (7–9). Among different assessments of nutritional status, the prognostic nutritional index (PNI), calculated based on the serum albumin concentration and peripheral blood lymphocyte count, is a reliable indicator of nutritional and immune status (9–11). Although PNI has been frequently applied in patients with malignancy or those during postoperative recovery (10, 12, 13), whether it is also an optimal indicator of the risk of CIN in patients receiving coronary angiography remains uncertain. Considering the small scale of previous studies (9, 11), we aimed at investigating the correlation of PIN with the risks of CIN and mortality in patients receiving angiography for CAD through incorporating the updated data in a meta-analysis of observational studies.

## 2. Methods

### 2.1. Study protocol

We reported our meta-analysis review in compliance with the Preferred Reporting Items for Systematic Reviews and Meta-Analyses (PRISMA) guidelines and the protocol was registered in PROSPERO (registration CRD42023389185).

### 2.2. Data sources and literature searches

We conducted a comprehensive search on Embase (Ovid interface), Medline (Ovid interface), Cochrane Library, and Google scholar from inception up to January 4 2023. The search terms including “prognostic nutritional index,” “coronary angiography,” “acute kidney injury” and their synonyms (e.g., percutaneous coronary intervention, PNI, and acute renal Insufficiency) were applied without language restriction. We used a combination of keywords and MeSH terms to enable a comprehensive search. The reference lists of eligible studies as well as systematic reviews were also screened to identify additional studies. Supplementary Table 1 summarized the search strategies for one of the databases.

After identification of eligible records, the titles and abstracts were reviewed initially by two independent reviewers to remove duplicate records and ineligible studies. Articles that fulfilled all the inclusion criteria were read independently in full-text by the same reviewers. Any disagreements were discussed until consensus was achieved.

### 2.3. Inclusion and exclusion criteria

Peer-reviewed articles including retrospective studies that met the following criteria were considered eligible: (a) studies that examined the relationship between PNI and CIN; (b) studies that measured PNI before the occurrence of CIN; (c) studies that focused on adults with CAD receiving coronary angiography or percutaneous coronary intervention (PCI); (d) availability of outcomes including the incidence of CIN and variables including PNI as binary or continuous variables, and (e) availability of full-texts.

Excluded studies were those (a) published as conference abstracts, editorials, letters, review articles, case series, and case reports; (b) focused on patients receiving cardiac surgery (e.g., coronary artery bypass graft) or a mixed population receiving PCI and surgical intervention; or (c) without providing data for risk calculation.

### 2.4. Data extraction

Two independent authors extracted the following data including details on patient information (e.g., patient population, age, body mass index, number of patients), PNI, publication year, country, outcomes (e.g., incidence of CIN), and other variables that may predict the risk of CIN (e.g., hypertension and smoking). Any disagreement was resolved through discussion between two independent authors. If necessary, we contacted the authors for missing data.

### 2.5. Outcomes

The definition of CIN was according to individual studies (e.g., serum creatinine concentrations and/or the presence of proteinuria). The primary outcome was the risk of CIN in patients with a low or high PNI, which was defined based on individual studies. If the PNI values were divided into more than two ranges in a study, only the associations (i.e., odds ratios) of CIN with the highest and the lowest ranges were used for analysis. Subgroup analysis based on the presence of ACS (i.e., yes vs. no) was performed. The secondary outcomes were the difference in PNI in patients with or without CIN as well as the associations of other variables (e.g., contrast volume and hypertension) with risk of CIN. The impacts of CIN occurrence or a low PNI on the risk of mortality in this patient population also served as secondary outcomes.

### 2.6. Assessment of quality of studies and certainty of evidence

The Newcastle-Ottawa Scale (NOS), which consisted of eight items (three domains: selection, comparability, and outcome), was used to investigate the quality for individual studies. For the selection and outcome domains, an item can be assigned a maximum of one star. On the other hand, each item in the comparability domain can be given a maximum of two stars. A study was considered to have a low risk of bias if more than seven stars were assigned. The certainty of evidence for each outcome was assessed independently by two authors based on the Grading of Recommendations Assessment, Development and Evaluation (GRADE) method (14). Any discrepancies regarding the quality of studies and certainty of evidence were settled by consulting a third author.

### 2.7. Statistical analysis

For dichotomous variables (e.g., hypertension) that may be linked to an increased risk of CIN, we calculated the odds ratio (OR) and the 95% confidence interval (CI). For continuous variables (e.g., age and contrast volume), the weighted mean difference (MD) and the corresponding CIs were reported. We used a random-effects model to conduct all analyses. Heterogeneity was considered statistically significant with the I squared value being >50%. Sensitivity analysis was conducted using a leave-one-out approach to confirm the reliability of outcomes. For publication bias, the risk was determined by visual assessment of a funnel plot if 10 or more studies reporting a particular outcome. The comprehensive Meta-Analysis V3 software (Biostat, Englewood, NJ, United States) or Review Manager were used for statistical analyses. A probability value (*p*) <0.05 was deemed statistically significant.

## 3. Results

### 3.1. Selection, characteristics, and quality of studies

**Figure 1:**
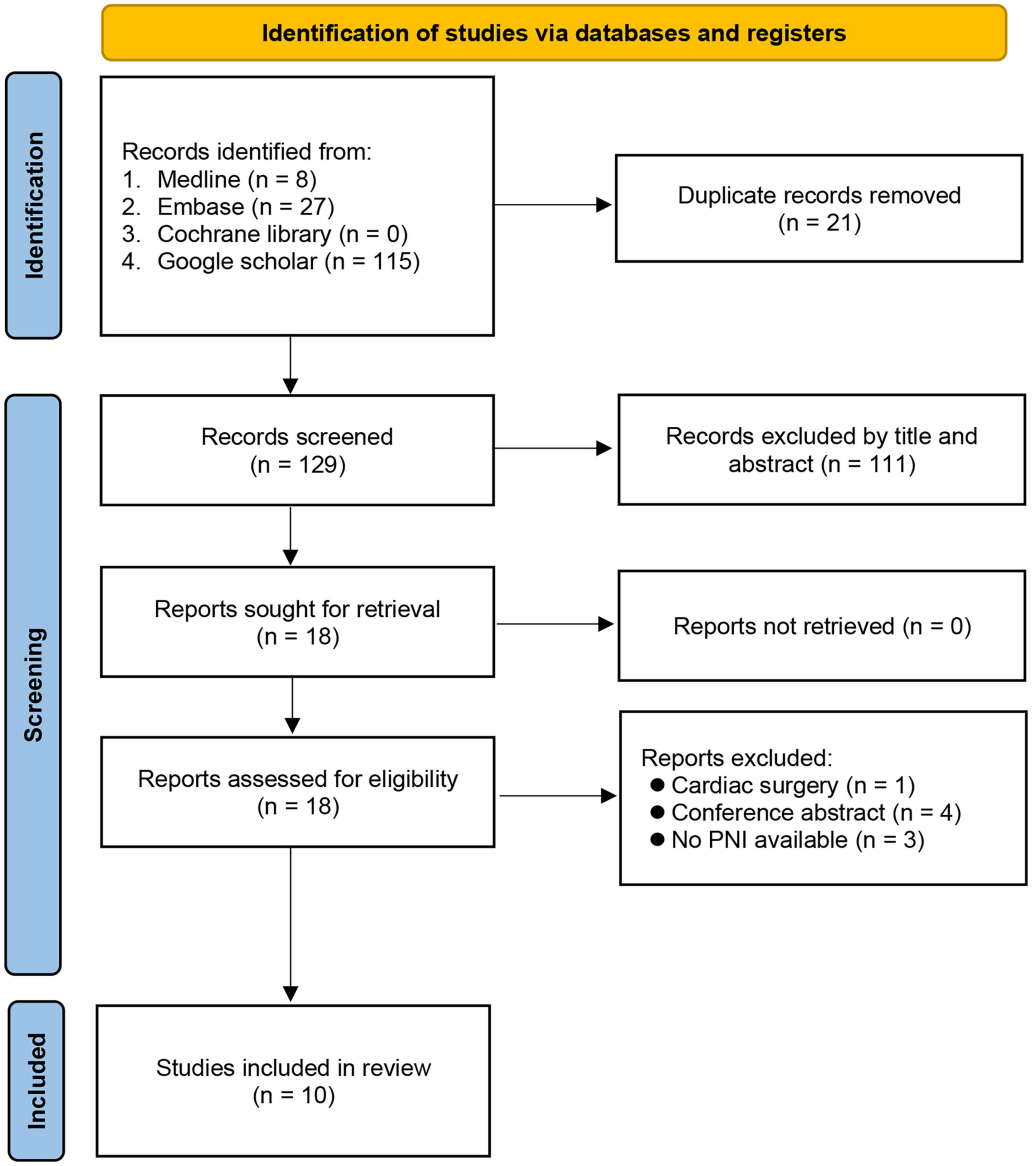
Flow chart of study selection. PNI: prognostic nutritional index.

Our initial database search yielded 150 records. After title and abstract screening, 111 non-relevant articles and 21 duplicate records were removed, giving 18 articles for full-text review. Eight studies were further excluded for the following reasons: (1) patients receiving cardiac surgery (*n* = 1); (2) articles presented as conference abstracts (*n* = 4); and (3) information related to PNI not provided (*n* = 3). Finally, a total of 10 studies with 17,590 patients were included for the current meta-analysis (Figure 1) (7, 9, 11, 15–21).

**Figure 2:**
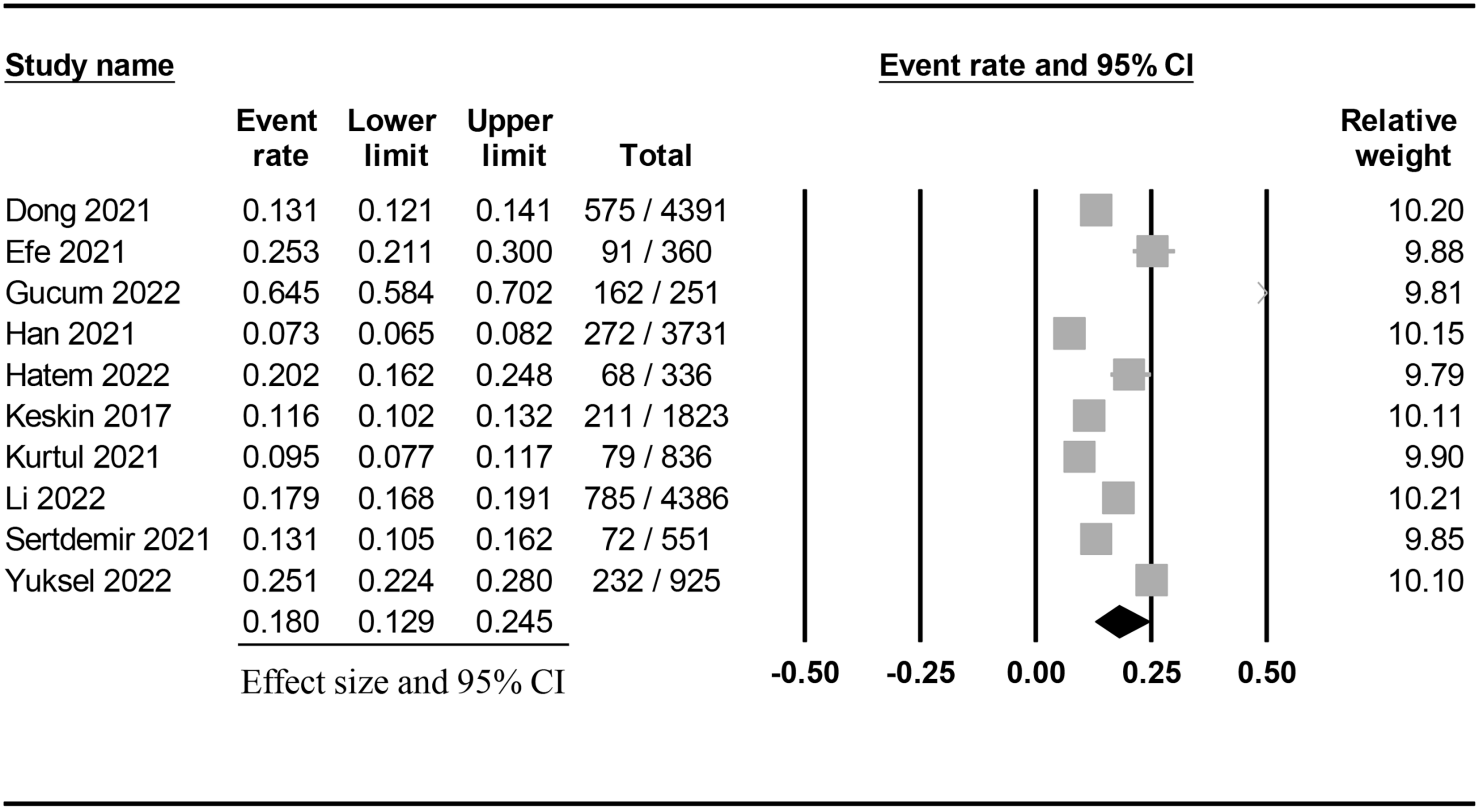
Pooled incidence of contrast-induced nephropathy following coronary angiography or percutaneous coronary intervention.

The characteristics of the included studies published between 2017 and 2022 are summarized in Table 1. All studies focused on adult patients (range of age: 55 to 72 years) receiving coronary angiography or percutaneous coronary intervention. The male proportion varied among the studies with a range of 54.4 to 84.1%. Six studies enrolled fewer than 1,000 patients (range: 251 to 925) (7, 11, 15, 17, 19, 20), while four recruited more than 1,000 patients (range: 1,823 to 4,391) (9, 16, 18, 21). All studies used circulating creatinine concentrations to determine the incidence of CIN in their participants. Nevertheless, the cutoff value of creatinine for defining CIN varied among the included studies (Supplementary Table 2). Only one study (16) provided data on the association of proteinuria with the development of CIN. The pooled incidence of CIN was 18% (95% CI: 12.9 to 24.5%) (Figure 2) with a range of 7.3 to 64.5% (Table 1). There was a trend of an association between a relatively higher incidence of CIN and small-scale studies (i.e., number of patients fewer than 1,000) (Table 1). Seven studies provided information on the risk of CIN in patients with a low and high PNI (7, 9, 11, 15, 18, 19, 21). The cut-off values for this binary variable are shown in Table 1. The ten included studies were conducted in three countries: Korea (*n* = 1) (16), China (*n* = 2) (9, 21), and Turkey (*n* = 7) (7, 11, 15, 17–20).

**Table 1:** Characteristics of studies (*n* = 10).

Study (year)	Population	Age (years)†	Male (%)†	BMI kg/m^2^†	N	PCI (%)†	AKI incidence (%)	PNI values for comparison	Country	NOS
Dong (2021) (9)	Patients were diagnosed as chronic kidney disease and CAD	70 vs. 68	72.2 vs. 73.6	–	4,391	73.7 vs. 77.1	13.1	<37.7 vs. >46.3	China	8
Efe (2021) (11)	Elderly patients over 65 years of age with CAD	72 vs. 69	61.5 vs. 63.9	29.3 vs. 28.7	360	33 vs. 25	25.3	<38 vs. >38	Turkey	6
Gucun (2022) (15)	Patients requiring nephrology consultation before CAG or PCI	70 vs. 67‡	67.1 vs. 69.4‡	–	251	–	64.5	<45 vs. >45	Turkey	5
Han (2021) (16)	Patients who received PCI	69 vs. 65	64.6 vs. 72.1	23.7 vs. 24.3	3,731	100 vs. 100	7.3	–	Korea	4
Hatem (2022) (17)	Patients over 18 years of age with NSTEMI and undergone CAG	65 vs. 60	63.2 vs. 75.4	27.5 vs. 27.3	336	95.6 vs. 73.5	20.2	–	Turkey	7
Keskin (2017) (18)	Patients with STEMI and undergone PCI	64 vs. 55‡	73.5 vs. 84.1‡	27.7 vs. 27.4‡	1823	–	11.6	<44 vs. >44	Turkey	9
Kurtul (2021) (19)	Patients with STEMI and treated with PCI	70 vs. 56	54.4 vs. 78.2	26 vs. 28	836	100 vs. 100	9.5	<38 vs. >38	Turkey	9
Li (2022) (21)	Patients undergoing CAG or PCI	69 vs. 67	60.1 vs. 67.3	–	4,386	46.6 vs. 45.2	17.9	<38 vs. >52	China	9
Sertdemir (2021) (20)	Patients diagnosed with ACS that underwent emergency PCI	65 vs. 62	59.7 vs. 63.5	27.9 vs. 27.5	551	100 vs. 100	13.1	–	Turkey	6
Yuksel (2022) (7)	Patients diagnosed as ACS who underwent early or primary PCI	69 vs. 60	75.5 vs. 67.6	28.6 vs. 27.9	925	100 vs. 100	25.1	<48.6 vs. >48.6	Turkey	7

† Presented as patients with acute kidney injury vs. those without acute kidney injury.

‡ Presented as low prognostic nutritional index vs. high prognostic nutritional index; AKI, acute kidney injury; CAG, coronary angiography; PCI, percutaneous coronary intervention; CAD, chronic coronary artery disease; NOS, Newcastle-Ottawa Scale.

The quality of studies based on NOS is demonstrated in Table 1. Six studies were considered to have a low risk of bias (range of total stars: 7–9) (7, 9, 17–19, 21), while four were judged to be of low quality (range of total stars: 4–6) (11, 15, 16, 20).

### 3.2. Outcomes

#### 3.2.1. Primary outcome

**Figure 3:**
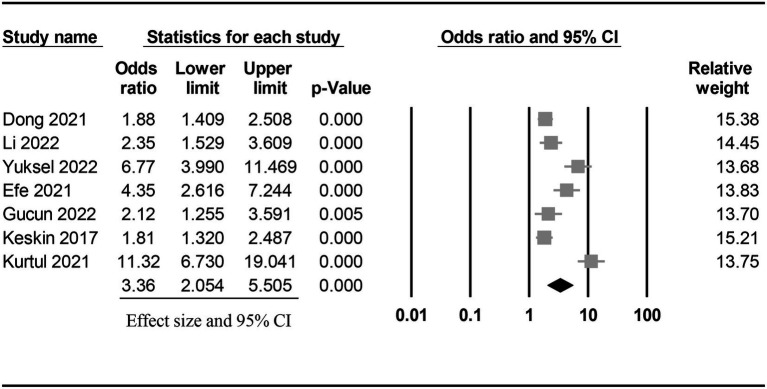
Forest plot showing the risk of contrast-induced nephropathy (CIN) in patients with a low and a high prognostic nutritional index (PNI). CI: confidence interval.

**Figure 4:**
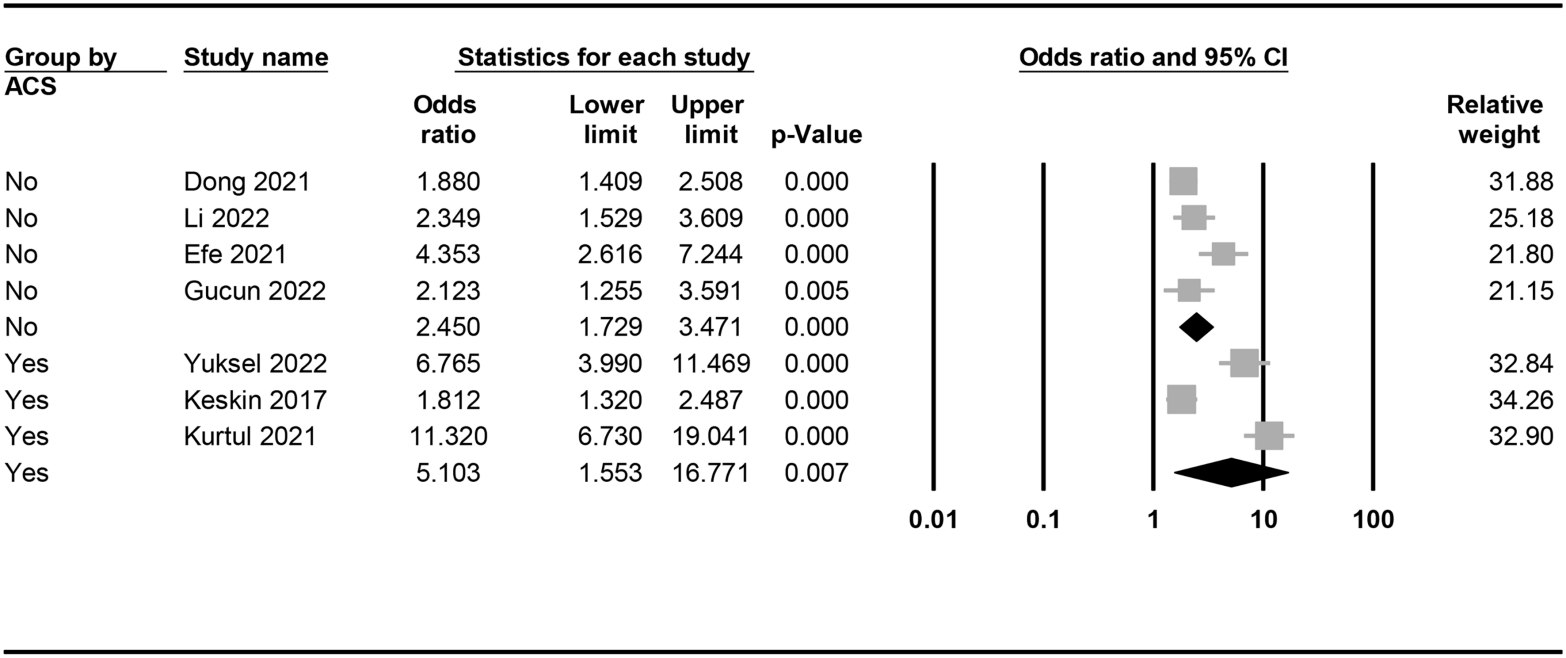
Subgroup analysis demonstrating the association between a low prognostic nutritional index (PNI) and the risk of developing contrast-induced nephropathy (CIN) in patients with or without acute coronary syndrome (ACS).

**Figure 5:**
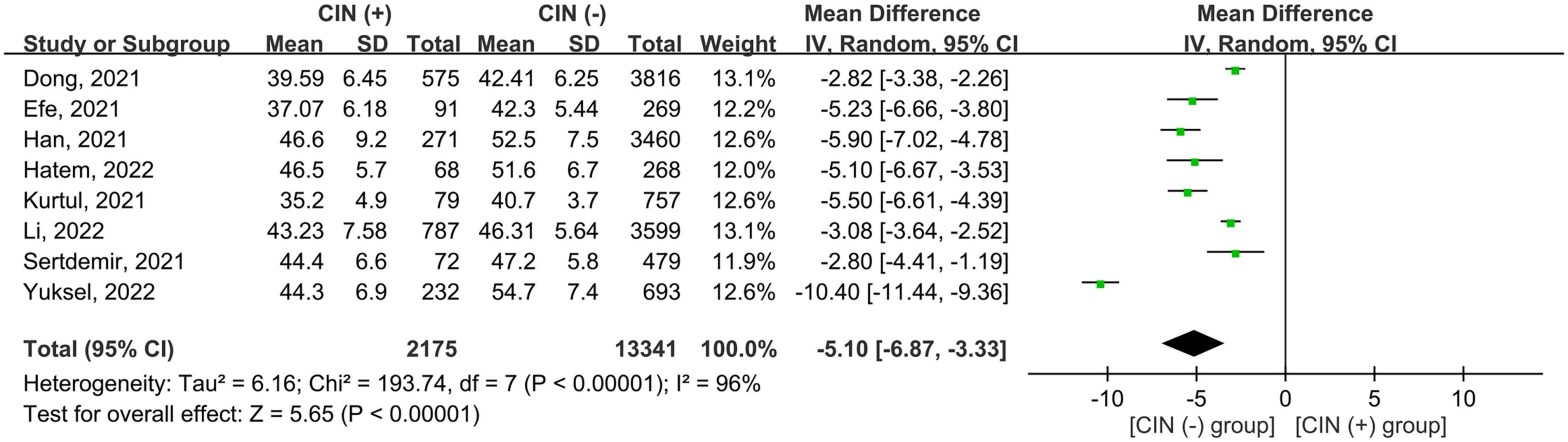
Forest plot showing the difference in prognostic nutritional index (PNI) between patients with and those without contrast induced nephropathy (CIN). CI, confidence interval; SD, standard deviation.

The risk of CIN in patients with a low or high PNI was available in seven studies (7, 9, 11, 15, 18, 19, 21). Meta-analysis of available data revealed a higher risk of CIN in patients with a low PNI compared to those with a high value (OR = 3.362, 95% CI: 2.054 to 5.505, *p* < 0.0001, *I*^2^ = 89.6%, seven studies, 12,972 patients, certainty of evidence: very low) (Figure 3). Sensitivity analysis showed a consistent finding. Subgroup analysis showed that the OR of CIN in patients without ACS was 2.45 (95% CI: 1.729 to 3.471), while the OR was 5.103 (95% CI:1.553 to 16.771) in those with ACS (Figure 4). To support this finding, our further analysis of the PNI in patients with or without CIN also demonstrated a lower PNI in patients with CIN compared to those without (MD = -5.1, 95% CI: −6.87 to −3.33, *p* < 0.00001, *I*^2^ = 96%, eight studies, 15,516 patients, sensitivity analysis: consistent; certainty of evidence: very low) (Figure 5) (7, 9, 11, 16, 17, 19–21).

#### 3.2.2. Secondary outcomes: Other risk factors

**Figure 6:**
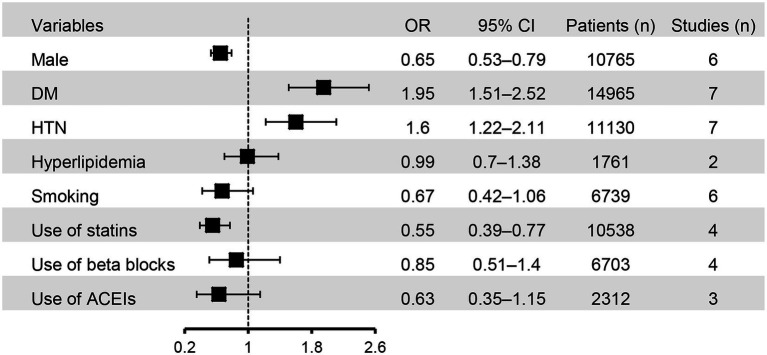
Forest plot showing the associations of binary variables with the risk of contrast induced nephropathy (CIN). HTN, hypertension; DM, diabetes mellitus; ACEIs, angiotensin-converting enzyme inhibitors; OR, odd ratio; CI, confidence interval.

The associations of binary variables with the risk of CIN are shown in Figure 6. Male gender (OR = 0.65, 95% CI: 0.53 to 0.79) and the use of statins (OR = 0.55, 95% CI: 0.39 to 0.77) were associated with a lower risk of CIN compared to female gender or those without using statins. In contrast, DM (OR = 1.95, 95% CI: 1.51 to 2.52) and hypertension (OR = 1.6, 95% CI: 1.22 to 2.11) were identified as risk factors for CIN. There was no significant association of hyperlipidemia, smoking, and the use of beta-blockers or angiotensin-converting enzyme inhibitors with the risk of CIN.

The correlations between continuous variables and the risk of CIN are summarized in Table 2. Patients with CIN were older (MD: 5.09 years, 95% CI: 3.2 to 6.99, *p* < 0.00001, 15,516 patients), had a higher creatinine level (MD: 0.33 mg/dL, 95% CI: 0.21 to 0.45, *p* < 0.00001, 10,794 patients), and received a higher contrast volume (MD: 11.33 mL, 95% CI: 2.82 to 19.83, *p* = 0.009, 10,214 patients) compared to those without CIN. On the other hand, albumin (MD: −2.87 g/L, 95% CI: −3.76 to −1.99, *p* < 0.00001, 14,344 patients), estimated glomerular filtration rate (MD: −17.16 mL/min/1.73m^2^, 95%CI: −25 to −9.32, *p* < 0.001, 14,820 patients), ejection fraction (MD: -5.6, 95%CI: −7.52 to −3.69, *p* < 0.00001, 7,394 patients), hemoglobin (MD: −0.93 mg/dL, 95% CI: −1.23 to −0.62, *p* < 0.00001, 10,289 patients), and lymphocyte (MD: –0.34 *10^9^/L, 95% CI: −0.6 to −0.09, *p* = 0.008, 14,344 patients) were found to be lower in patients with CIN than in those without (Table 2). The certainty of evidence for these outcomes was considered to be very low.

**Table 2:** Association of continuous variables with risk of contrast induced nephropathy.

Variable	Studies	Participants	MD	95% CI	*p* value	*I* ^2^	Sensitivity analysis	Certainty of evidence
Age	8	15,516	5.09	3.2 to 6.99	<0.00001	93%	Consistent	⨁◯◯◯ Very Low
BMI	5	6,379	−0.37	−1.19 to 0.45	0.37	78%	Consistent	⨁◯◯◯ Very Low
Albumin	6	14,344	−2.87	−3.76 to −1.99	<0.00001	88%	Consistent	⨁◯◯◯ Very Low
Creatinine	6	10,794	0.33	0.21 to 0.45	<0.00001	94%	Consistent	⨁◯◯◯ Very Low
eGFR	6	14,820	−17.16	−25 to −9.32	<0.001	98%	Consistent	⨁◯◯◯ Very Low
Ejection fraction	6	7,394	−5.6	−7.52 to −3.69	<0.00001	82%	Consistent	⨁◯◯◯ Very Low
CRP	5	9,649	0.58	−1.37 to 2.53	0.56	98%	Inconsistent	⨁◯◯◯ Very Low
Hemoglobin	6	10,289	−0.93	−1.23 to −0.62	<0.00001	80%	Consistent	⨁◯◯◯ Very Low
Lymphocyte	6	14,344	−0.34	−0.6 to −0.09	0.008	98%	Consistent	⨁◯◯◯ Very Low
Contrast volume	5	10,214	11.33	2.82 to 19.83	0.009	69%	Consistent	⨁◯◯◯ Very Low

BMI, body mass index; MD, mean difference; CRP, C-reactive protein, eGFR, estimated glomerular filtration rate; CI, confidence interval.

#### 3.2.3. Secondary outcomes: Impacts of CIN and low PNI on mortality risk

**Figure 7:**
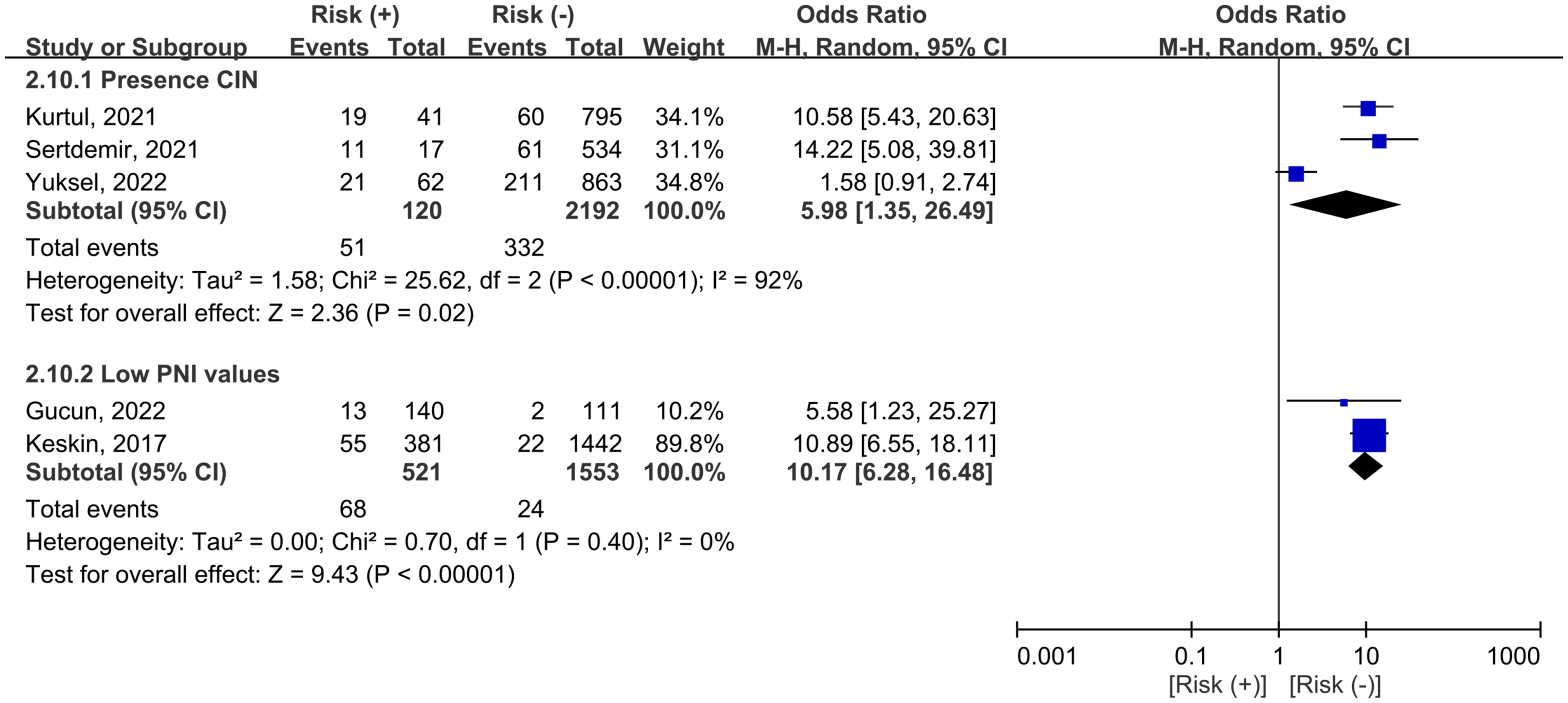
Forest plot showing the associations of contrast induced nephropathy (CIN) or a low prognostic nutritional index (PNI) with the risk of mortality. CI, confidence interval.

Three and two studies reported the association of CIN or a low PNI with the risk of mortality, respectively (Figure 7). The occurrence of CIN was related to an increased risk of mortality in adult patients receiving percutaneous coronary intervention or coronary angiography (OR = 5.98, 95% CI: 1.35 to 26.49, *p* = 0.02, *I*^2^ = 92%, 2,312 patients) (7, 19, 20). Similarly, the presence of a low PNI was also associated with an increased risk of mortality (OR = 10.17, 95% CI: 6.28 to 16.48, *p* < 0.00001, *I*^2^ = 0%, 2074 patients) (15, 18).

## 4. Discussion

This meta-analysis involving 17,590 patients showed a higher risk of CIN in patients with a low PNI than in those with a high value. Sensitivity analyses also demonstrated consistent findings. Importantly, the occurrence of CIN was linked to a higher risk of mortality in patients receiving either coronary angiography or intervention. Likewise, a lower PNI correlated with a higher risk of mortality. Regarding the other risk factors associated with CIN, patients who were males or those using statins presented with a lower risk of CIN compared to females or those without statin use. Similar to previously reported findings (3), the current study demonstrated that older age, DM, hypertension, a higher baseline creatinine level as well as a higher volume of contrast medium positively correlated with the development of CIN. In addition, among patients with ACS, the risk of a low PNI on the development of CIN was even more significant. Taken together, our findings provided robust evidence that a low PNI may be a specific warning sign of the subsequent development of CIN after coronary angiography or interventions. Moreover, both a low PNI and the occurrence of CIN may contribute to an increased mortality rate.

Given that CIN is the third largest cause of acquired kidney injury and is related to a poor long-term outcome, identifying potentially reversible risk factors is pivotal (3, 22). As malnutrition is commonly observed in patients with CAD and is closely associated with kidney injury, a prompt evaluation of nutritional status is necessary, especially in individuals exposed to nephrotoxic materials such as contrast medium (9, 11). A recently released meta-analysis of 26 studies focusing on the correlation of CIN with inflammatory Indicators and hematological indices involving over 29,000 patients who underwent coronary intervention found that parameters including C-reactive protein, lymphocyte ratio, neutrophil to lymphocyte ratio, hematocrit, and red blood cell distribution width were weakly associated with CIN (23). In contrast, the current study used PNI as an index to incorporate serum albumin concentration and peripheral blood lymphocyte count so that both the nutritional and immune status could be readily assessed. Our findings highlighted that PNI may be an optimal indicator of the subsequent development of CIN in patients receiving either coronary angiography or intervention.

Although the pathophysiology of CIN remains unclear, direct toxicity of contrast medium on renal capillaries and tubules as well as the generation of reactive oxygen species are proposed to be the two major mechanisms (3, 24). A number of *in vitro* studies have demonstrated cell death or apoptosis of endothelial and renal tubular epithelial cells on exposure to contrast medium (24–26). Likewise, *in vivo* studies have also shown a contrast medium-induced decrease in oxygenation and nitric oxide levels as well as an accumulation of ROS in renal medullary, resulting in renal functional impairment (3, 27, 28). In addition, signal transduction pathways including Akt and ERK1/2 that enable cells to overcome stress and to proliferate have been observed to be suppressed on exposure to iodine, the major component of contrast medium (29). Although hydration, N-acetylcysteine, and statins have been proposed for the prevention of CIN, the strength of clinical evidence was weak (30–32). The lack of an effective prophylactic strategy (3, 29) further underscored the importance of identifying patients who are at high risk of CIN development.

The contributions of malnutrition to CIN are multi-faceted. First, a previous systematic review has reported an association of malnutrition with a reduced glomerular filtration rate, renal plasma flow as well as an impaired ability to concentrate urine and excrete the osmotic load in children and adults (33). Second, malnutrition can affect the body’s ability to maintain fluid balance (34), which is important for sustaining adequate renal perfusion during imaging procedures that involve contrast dyes. Third, because certain micronutrients, such as vitamins C and E, have been shown to be protective against CIN (35, 36), deficiencies of these nutrients in diet may predispose to this disorder. Fourth, a retrospective study involving 2,989 patients with cardiovascular disease reported a higher proportion of individuals with estimated glomerular filtration rate (eGFR) <60 min/mL in those with a low lean mass index compared to those with a high index (37), implicating a potential correlation between a low muscularity and an impaired baseline renal function. In summary, as pre-procedural renal function impairment is known to increase the susceptibility to CIN (3), current evidence suggests that a lack of proper nutrition may increase the risk of developing this serious kidney condition despite the need for more research to fully understand the association.

Several clinical risk factors have been found to be associated with the development of CIN. In concert with our findings, pre-existent renal insufficiency, DM, older age, acute myocardial infarction, and the volume of contrast medium have been reported to link to the occurrence of CIN (3). Interestingly, previous studies have shown an impact of gender on the progression of kidney disease (38). Despite the demonstration of a consistent protective effect on the development of kidney damage after ischemia–reperfusion injury in females in experimental settings (39, 40), the female gender has been identified as a risk factor in clinical studies developed to predict the risk of acute kidney injury after cardiac surgeries, aminoglycoside nephrotoxicity, and CIN (41). Likewise, although a previous meta-analysis of 28 studies including over 6 million patients on the effect of gender on the risk of hospital-associated acute kidney injury revealed that hospitalized men were more likely to develop acute kidney injury which required dialysis than hospitalized women (42), another clinical observable study involving 2,851 patients showed a 1.42 fold increase in risk of CIN among females compared to males who received coronary angiography or percutaneous coronary intervention (41). Therefore, the actual role of sex hormones in the pathophysiology of CIN remains to be elucidated. On the other hand, CIN has been most frequently observed in patients receiving coronary interventions for ACS (43, 44) probably attributable to the high probability of ACS-related hemodynamic instability and the resulting organ hypoperfusion (43). A clinical study recruiting 1,300 consecutive AMI patients undergoing angiography suggested that CIN may positively correlate with long-term mortality in patients with ACS irrespective of its definitions (44). In addition to the conventional risk factors for CIN, our findings highlighted a probable role of malnutrition in this clinical setting.

This study had its limitations. First, the inclusion of only observational studies in this meta-analysis due to a lack of randomized controlled trials may attenuate the power to investigate the causality between PNI and CIN. Second, the variation in definitions of CIN across the included studies may contribute to heterogeneity of the studied population. Nevertheless, most of the studies defined CIN as an increase in serum creatinine ≥0.3 mg/dL or ≥ 50% from the baseline in a short duration after coronary angiography or intervention (45). Moreover, because only one study (16) provided data on the incidence of proteinuria, the link between proteinuria and CIN could not be analyzed in the present study. Third, the incidence of CIN of the included studies ranged widely from 7.3 to 64.5% with a trend of an increased incidence of CIN in small-scale studies, highlighting the possibility of selection bias. To address this concern, we conducted sensitivity analysis with a leave-one-out approach, which consistently showed an inverse correlation between PNI and the risk of CIN. Fourth, although pre-existing renal insufficiency is a well-known risk factor for CIN (3), most studies only provided data on the mean baseline eGFR of their participants and did not categorize them according to their eGFR to enable a subgroup analysis. Therefore, the association between PNI and CIN based on baseline eGFR could not be assessed in the present study. Finally, because only one study reported a higher incidence of renal replacement therapy in patients with a low PNI (i.e., <45) compared to those with a high PNI (i.e.,>45) (15), the correlation of PNI values with the incidence of more severe forms of CIN could not be elucidated.

## 5. Conclusion

The results of this meta-analysis showed that patients with a low prognostic nutritional index who underwent coronary angiography or intervention were associated with an increased risk of contrast-induced nephropathy. Both a low prognostic nutritional index and the development of contrast-induced nephropathy were associated with an elevated mortality. Older age, female gender, diabetes, hypertension, renal impairment at baseline, a higher volume of contrast medium, and a status of acute coronary syndrome were significant risk factors for contrast-induced nephropathy. Therefore, besides conventional risk factors, malnutrition may be an important contributor to the risk of contrast-induced nephropathy. Further randomized controlled trials are necessary to support our findings.

## Data availability statement

The original contributions presented in the study are included in the article/Supplementary material, further inquiries can be directed to the corresponding author.

## Author contributions

W-TC and C-KS: conceptualization. J-YW and P-YH: methodology and software. T-HL and F-CK: validation. KCH and F-CK: formal analysis. W-TC, Y-JC, and C-KS: investigation. J-YW and Y-TL: resources. W-TC and K-CH: data curation and writing—review and editing. W-TC, K-CH, and C-KS: writing—original draft preparation. K-CH: visualization and supervision. All authors have read and agreed to the published version of the manuscript.

## Conflict of interest

The authors declare that the research was conducted in the absence of any commercial or financial relationships that could be construed as a potential conflict of interest.

## Publisher’s note

All claims expressed in this article are solely those of the authors and do not necessarily represent those of their affiliated organizations, or those of the publisher, the editors and the reviewers. Any product that may be evaluated in this article, or claim that may be made by its manufacturer, is not guaranteed or endorsed by the publisher.
