# Supplementary material for: Association of prognostic nutritional index with risk of contrast induced nephropathy: A meta-analysis
Source: Front Nutr. 2023 Mar 23;10:1154409. doi: 10.3389/fnut.2023.1154409 (PMC10076581; doi:10.3389/fnut.2023.1154409)
Supplement: Supplementary file 1 [file Data_Sheet_1.docx]

**Supplemental Table 1.** Search strategies for Medline

| Database | # | Search syntax |
| --- | --- | --- |
| **MEDLINE** | 1 | ("PCI" OR "Left Main Disease" OR "Coronary Angiograph*" OR "coronary artery disease" OR "Percutaneous Coronary Intervention" OR "Myocardial infarction" OR "MI").mp |
|  | 2 | exp "Percutaneous Coronary Intervention"/ or exp "coronary artery disease"/ or exp "Coronary Angiography"/ |
|  | 3 | ("Prognostic nutritional index" OR "Prognostic Nutritional Indices" OR "PNI").mp |
|  | 4 | ("CI-AKI" OR "CA-AKI" OR "Contrast-induced acute kidney injury" OR "Contrast-Associated Acute Kidney Injury" OR "Contrast-Induced Nephropathy" OR "Contrast-Associated Nephropathy" OR "Acute Renal Insufficiency" OR "Acute Kidney Insufficiency" OR "Renal Insufficienc*" OR "Kidney insufficienc*" OR "Acute kidney injury" OR "AKI" OR "Nephropathy").mp |
|  | 5 | exp "Acute Kidney Injury"/ OR exp "Renal Insufficiency"/ |
|  | 6 | (1 OR 2) AND (3) AND (4 OR 5) |

**Supplemental Table 2.** Definition of contrast-induced nephropathy

| Study  (year) | Contrast-induced nephropathy |
| --- | --- |
| Dong (2021) | Creatinine increase ≥0.3 mg/dL or 50% from baseline within the first 48 hours following CAG |
| Efe  (2021) | An increase of 0.5 mg/dL in plasma creatinine levels or a 25% increase in basal creatinine within 72 hours after the procedure recommended by the European Society of Urogenital Radiology (ESUR). |
| Gucun (2022) | Within the 72 hours following intravascular contrast media administration; an absolute rise of 0.5 mg/dL (44 μmol/L), or a relative 25% rise from the baseline, in serum creatinine value, in the absence of other causes was defined as CA-AKI |
| Han  (2021) | CA-AKI was defined as a rise in serum creatinine ≥ 0.3-or 1.5 than the baseline value within 48 h after PCI according to KDIGO guidelines. |
| Hatem (2022) | CA-AKI was defined as a 25% or .3 mg/dL increase in baseline serum creatinine levels within 48–72 h after coronary angiography |
| Keskin (2017) | CA-AKI was defined as an increase in the serum creatinine level to ≥0.3 mg/dL or a relative increase in the serum creatinine level by ≥ 50%. |
| Kurtul (2021) | CA-AKI was defined as an increase in the plasma creatinine level of at least 0.5 mg per deciliter or at least a 25% increase from the baseline level within 3 days after exposure to contrast material due to primary PCI. |
| Li  (2022) | an increase in serum creatinine by more than 44 μmol/L (0.5 mg/dl) or 25%; b. within 72 h of intravascular contrast injection; c. no alternative etiology. |
| Sertdemir (2021) | increase in serum creatinine (SCr) ≥0.3 mg/dL or ≥50% from the baseline SCr levels within 48 h after PCI. |
| Yuksel (2022) | CA-AKI was assumed to have developed when creatinine level was >.5 mg/dl or increased by 25%. |

CA-AKI= Contrast-associated acute kidney injury; PCI = percutaneous coronary intervention; CAG=coronary angiography
